# Supplementary material for: PRIME-3D2D is a 3D2D model to predict binding sites of protein–RNA interaction
Source: Commun Biol. 2020 Jul 16;3:384. doi: 10.1038/s42003-020-1114-y (PMC7366699; doi:10.1038/s42003-020-1114-y)
Supplement: Supplementary file 1 — Supplementary Information [file 42003_2020_1114_MOESM1_ESM.pdf]

PRIME-3D2D is a 3D2D model to predict binding sites of protein-RNA interaction

Juan Xie[1], Jinfang Zheng[1], Xu Hong, Xiaoxue Tong and Shiyong Liu*

School of Physics, Huazhong University of Science and Technology, Wuhan, Hubei 430074, China

* To whom correspondence should be addressed. Email: liushiyong@gmail.com

[1]These authors contributed equally to this work

Key words: protein-RNA interactome, 3D2D-model, alignment, binding sites

Figures

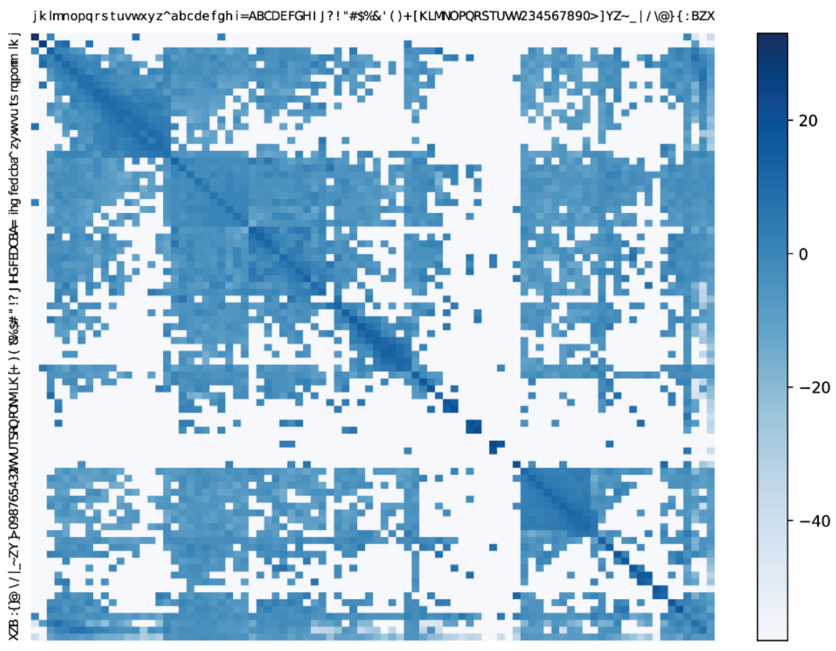

Supplementary Fig. 1. *RNABLOSUM80 matrix.* It can be download from http://www.rnabinding.com/PRIME-3D2D/help.html.

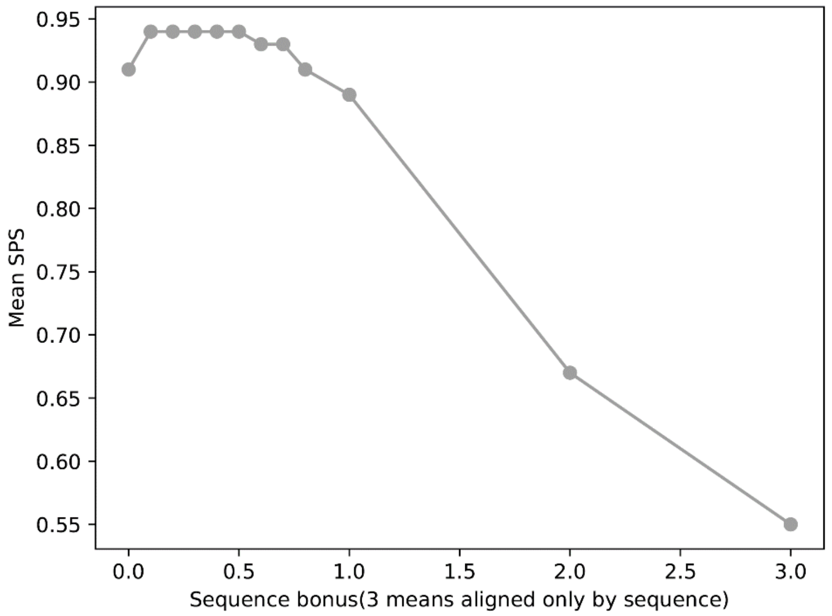

Supplementary Fig. 2. *Mean SPS vs different sequence bonus testing inBRAliBase II*. The scoring matrix is calculated by RNABLOSUM80 + bonus * NUC.4.4. For bonus = 3.0, the scoring matrix is NUC.4.4. The figure shows bonus = 0.1, 0.2, 0.3, 0.4, 0.5 achieves the best Mean SPS. In RNA2dA, the bonus is determined as 0.2.

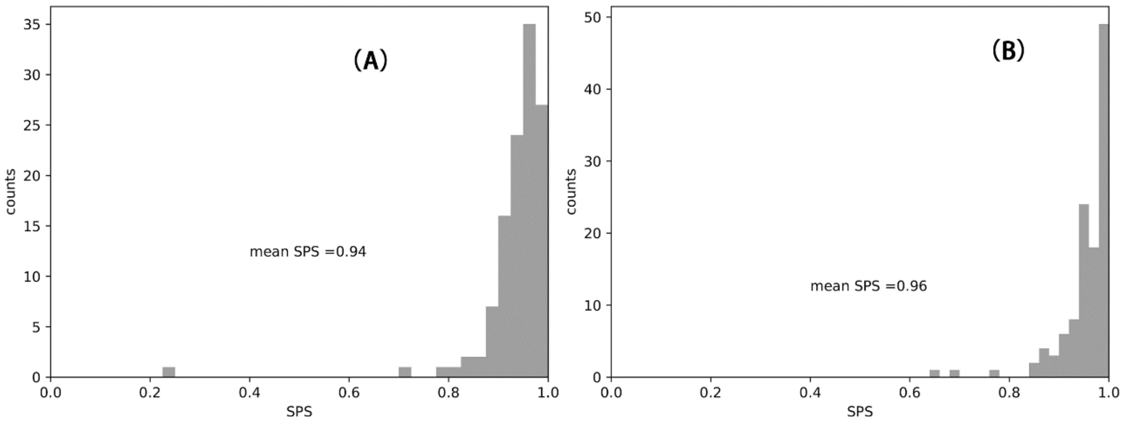

Supplementary Fig. 3. *The distribution of SPS for RNA2dA (A) and LocARNA (B) benchmarking in BRAliBase II.*

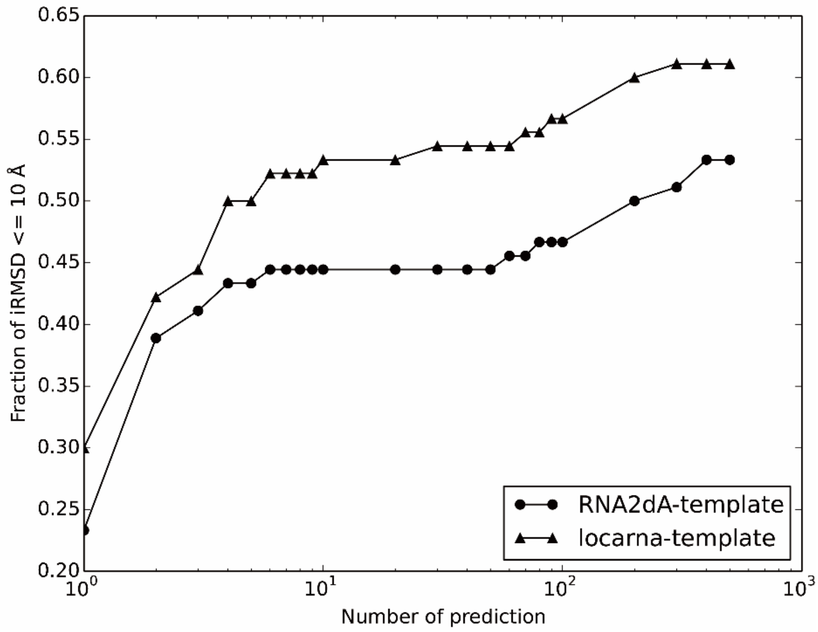

Supplementary Fig. 4. *Searching templates in NRBC439.* The fraction of iRMSD <= 10 Å was plotted against the number of prediction, in NRBC90 as targets vs NRBC349 as templates. iRMSD is used to distinguish similar (iRMSD <= 10Å) or non-similar binding mode (iRMSD >=10 Å). The fraction of iRMSD <= 10Å is defined as that the number of targets which can detected a template of iRMSD <= 10Å is divided by 90. Templates were sorted by TM-score. The fraction of iRMSD <= 10Å using LocARNA as the RNA alignment is better than using RNA2dA, suggesting that a method combining RNA primary/secondary structural information performed better than that using RNA secondary structure alone.

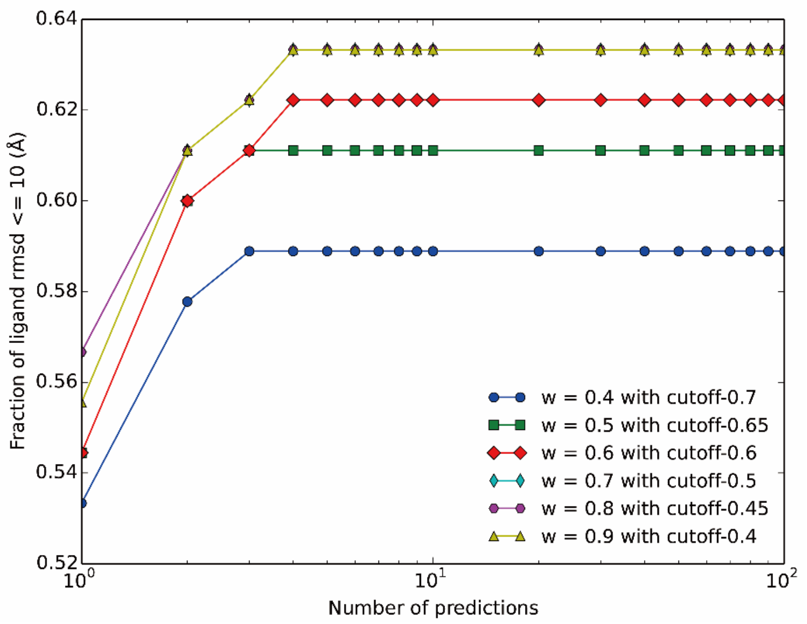

Supplementary Fig. 5. *Determining the weight of template-based protein-RNA structure prediction*. Targets (90 newer complexes) were predicted by 3D2D-PRIME using NRBC349 as templates. The models are ranked by 3D2D-score (using different weights). The docking of a target is prediction as successful if at least one model is built successfully within a set number of predictions (ligand RMSD between predicted model and native structure is less than 10Å). W = X with cutoff-Y means that model is built by template with a 3D2D-score (weight X) above the transition points cutoff -Y.

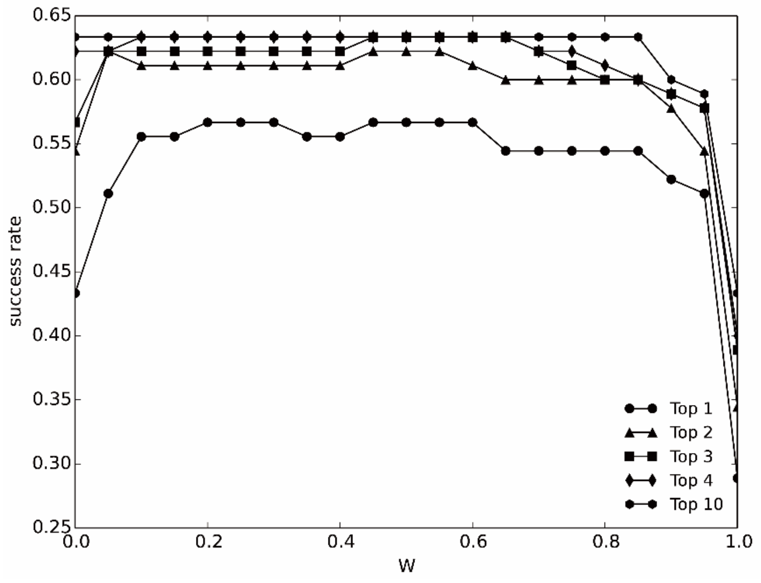

Supplementary Fig. 6. *Success rate vs weight with top N prediction in benchmarking of PRIME-3D2D.* Targets (90 newer complexes) were predicted using templates (349 older complexes). The models are ranked separately by 3D2D-score. The docking of a complex was successful if at least one prediction within a set number of predictions was successful. X-axis stands for the different weight between protein structural similarity and RNA SSI. This figure shows a similarity score combining protein similarity score and RNA SSI can select more correct models.

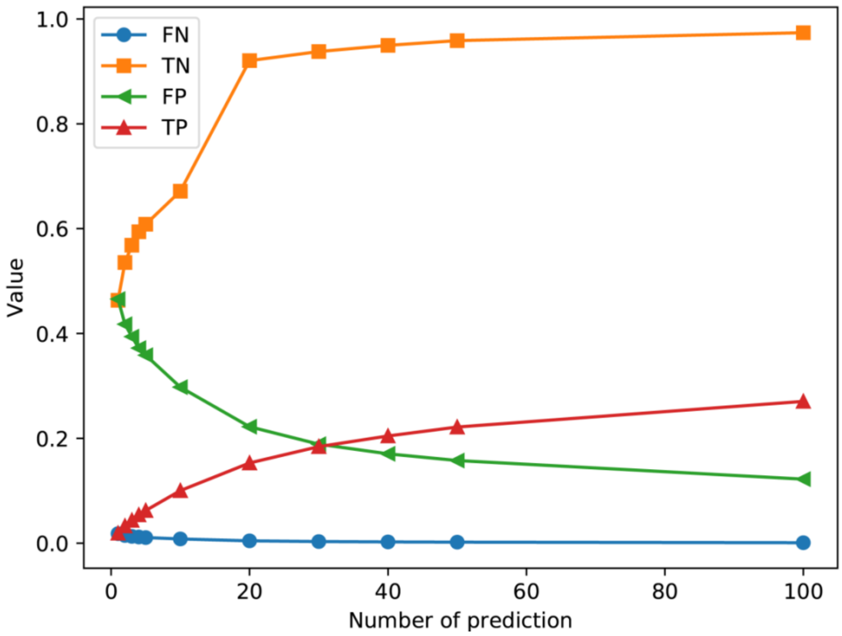

Supplementary Fig. 7. *RNA and protein binding sites prediction using NRBC439 as templates and yeast interactome as target*. TP, TN, FP, FN are plotted against the number of predictions, in comparison of NRBC439 vs yeast interactome. The 3D2D-models are sorted by 3D2D-score.
