# Supplementary material for: PRIME-3D2D is a 3D2D model to predict binding sites of protein–RNA interaction
Source: Commun Biol. 2020 Jul 16;3:384. doi: 10.1038/s42003-020-1114-y (PMC7366699; doi:10.1038/s42003-020-1114-y)
Supplement: Supplementary file 2 — Reporting Summary [file 42003_2020_1114_MOESM2_ESM.pdf]

nature research

Corresponding author(s): Shiyong Liu

Last updated by author(s): Jun 16, 2020

Reporting Summary

Nature Research wishes to improve the reproducibility of the work that we publish. This form provides structure for consistency and transparency in reporting. For further information on Nature Research policies, see our Editorial Policies and the Editorial Policy Checklist.

Statistics

For all statistical analyses, confirm that the following items are present in the figure legend, table legend, main text, or Methods section.

n/a	Confirmed	
☐	☒	The exact sample size (n) for each experimental group/condition, given as a discrete number and unit of measurement
☒	☐	A statement on whether measurements were taken from distinct samples or whether the same sample was measured repeatedly
☐	☒	The statistical test(s) used AND whether they are one- or two-sided<br>*Only common tests should be described solely by name; describe more complex techniques in the Methods section.*
☐	☒	A description of all covariates tested
☐	☒	A description of any assumptions or corrections, such as tests of normality and adjustment for multiple comparisons
☐	☒	A full description of the statistical parameters including central tendency (e.g. means) or other basic estimates (e.g. regression coefficient) AND variation (e.g. standard deviation) or associated estimates of uncertainty (e.g. confidence intervals)
☒	☐	For null hypothesis testing, the test statistic (e.g. F, t, r) with confidence intervals, effect sizes, degrees of freedom and P value noted<br>*Give P values as exact values whenever suitable.*
☒	☐	For Bayesian analysis, information on the choice of priors and Markov chain Monte Carlo settings
☐	☒	For hierarchical and complex designs, identification of the appropriate level for tests and full reporting of outcomes
☐	☒	Estimates of effect sizes (e.g. Cohen's d, Pearson's r), indicating how they were calculated

Our web collection on statistics for biologists contains articles on many of the points above.

Software and code

Policy information about availability of computer code

Data collection	1. PDB data. The benchmarked data NRBC439 was downloaded from http://www.rnabinding.com/PRIME.html. And the independent test set RB75, RB172 and RB344 were downloaded from https://www.sciencedirect.com/science/article/pii/S1047847711002851?via%3Dihub, https://www.ncbi.nlm.nih.gov/pmc/articles/PMC3962366/#!po=5.00000 and https://bmcbioinformatics.biomedcentral.com/articles/10.1186/s12859-015-0691-0/tables/4, respectively.<br>2. Genome data. The CLIP-seq data was downloaded from CLIPdb. And the experimentally determined RNA secondary structure data was download from https://www.ncbi.nlm.nih.gov/pubmed/23064747.<br>3. BRAliBase II was download from https://www.ncbi.nlm.nih.gov/pubmed/15860779.
Data analysis	The webserver of PRIME-3D2D was present in http://www.rnabinding.com/PRIME-3D2D/.

For manuscripts utilizing custom algorithms or software that are central to the research but not yet described in published literature, software must be made available to editors and reviewers. We strongly encourage code deposition in a community repository (e.g. GitHub). See the Nature Research guidelines for submitting code & software for further information.

Data

Policy information about availability of data

All manuscripts must include a data availability statement. This statement should provide the following information, where applicable:

- Accession codes, unique identifiers, or web links for publicly available datasets
- A list of figures that have associated raw data
- A description of any restrictions on data availability

1. PDB data. The benchmarked data NRBC439 was downloaded from http://www.rnabinding.com/PRIME.html. And the independent test set RB75, RB172 and RB344 were downloaded from https://www.sciencedirect.com/science/article/pii/S1047847711002851?via%3Dihub, https://www.ncbi.nlm.nih.gov/pmc/articles/

PMC3962366/#!po=5.00000 and https://bmcbioinformatics.biomedcentral.com/articles/10.1186/s12859-015-0691-0/tables/4, respectively.
2. Genome data. The CLIP-seq data was downloaded from CLIPdb. And the experimentally determined RNA secondary structure data was download from https://www.ncbi.nlm.nih.gov/pubmed/23064747.
3. BRAliBase II was download from https://www.ncbi.nlm.nih.gov/pubmed/15860779.3.
Figure1 used the RB75, RB172 and RB344 data and genome data; Figure S2, Figure S3 used the data BRAliBase II; Figure S4, Figure S5, Figure S6 and Figure S7 used the NRBC349; Figure S8, Figure9 and Figure 10 used the genome data.

Field-specific reporting

Please select the one below that is the best fit for your research. If you are not sure, read the appropriate sections before making your selection.

☒ Life sciences ☐ Behavioural & social sciences ☐ Ecological, evolutionary & environmental sciences

For a reference copy of the document with all sections, see nature.com/documents/nr-reporting-summary-flat.pdf

Life sciences study design

All studies must disclose on these points even when the disclosure is negative.

Sample size	Yeast genome
Data exclusions	In order to avoid the redundancy of the results, NRBC439 was deduplicates according to the RNA; when searching for templates, the structure using itself as the template is removed. Yeast genomic data, excluding the sequences without experimental RNA secondary structure.
Replication	Calculation results can be reproduced.
Randomization	When using NRBC439 to test the ability of LocARNA and RNA2da in searching templates , 80% with an older deposit date are designated as the templates (NRBC349), and 20% with a newer deposit date are designated as targets (NRBC90).
Blinding	The author is informed about the data and results

Reporting for specific materials, systems and methods

We require information from authors about some types of materials, experimental systems and methods used in many studies. Here, indicate whether each material, system or method listed is relevant to your study. If you are not sure if a list item applies to your research, read the appropriate section before selecting a response.

Materials & experimental systems

n/a	Involved in the study
☒	☐ Antibodies
☒	☐ Eukaryotic cell lines
☒	☐ Palaeontology and archaeology
☒	☐ Animals and other organisms
☒	☐ Human research participants
☒	☐ Clinical data
☒	☐ Dual use research of concern

Methods

n/a	Involved in the study
☒	☐ ChIP-seq
☒	☐ Flow cytometry
☒	☐ MRI-based neuroimaging
